# Redefining the Expression and Function of the Inhibitor of Differentiation 1 in Mammary Gland Development

**DOI:** 10.1371/journal.pone.0011947

**Published:** 2010-08-03

**Authors:** Radhika Nair, Simon Junankar, Sandra O'Toole, Jaynish Shah, Alexander D. Borowsky, J. Michael Bishop, Alexander Swarbrick

**Affiliations:** 1 Cancer Research Program, Garvan Institute of Medical Research, Darlinghurst, New South Wales, Australia; 2 Department of Tissue Pathology and Diagnostic Oncology, Royal Prince Alfred Hospital, Camperdown, New South Wales, Australia; 3 Clinical School, Sydney Medical School, Sydney University, Sydney, New South Wales, Australia; 4 Department of Biochemistry and Molecular Medicine, University of California Davis, Sacramento, California, United States of America; 5 G.W. Hooper Research Foundation, University of California San Francisco, San Francisco, California, United States of America; 6 St Vincent's Clinical School, University of New South Wales, Kensington, New South Wales, Australia; University Medical Center Utrecht, Netherlands

## Abstract

The accumulation of poorly differentiated cells is a hallmark of breast neoplasia and progression. Thus an understanding of the factors controlling mammary differentiation is critical to a proper understanding of breast tumourigenesis. The Inhibitor of Differentiation 1 (Id1) protein has well documented roles in the control of mammary epithelial differentiation and proliferation *in vitro* and breast cancer progression *in vivo*. However, it has not been determined whether Id1 expression is sufficient for the inhibition of mammary epithelial differentiation or the promotion of neoplastic transformation *in vivo*. We now show that Id1 is not commonly expressed by the luminal mammary epithelia, as previously reported. Generation and analysis of a transgenic mouse model of Id1 overexpression in the mammary gland reveals that Id1 is insufficient for neoplastic progression in virgin animals or to prevent terminal differentiation of the luminal epithelia during pregnancy and lactation. Together, these data demonstrate that there is no luminal cell-autonomous role for Id1 in mammary epithelial cell fate determination, ductal morphogenesis and terminal differentiation.

## Introduction

In many cancers such as breast cancer, the degree of differentiation correlates inversely with cancer grade and patient mortality. Many canonical oncogenes and tumour suppressors have roles in differentiation, such as Notch and Wnt [1], Hedgehog [2], Rb [3] and BRCA1 [4].Thus an analysis of the genes controlling mammary differentiation may lead to insights into the factors and mechanisms controlling breast tumourigenesis. The Id family of transcriptional regulators, composed of Id1, Id2, Id3 and Id4 belong to the basic helix-loop-helix (bHLH) family of transcription factors. Unlike other family members, Id proteins lack DNA binding domains and thus act as dominant negative inhibitors of other transcription factors, including members of the HLH and Ets families (reviewed in [5]). By binding to these factors, they prevent the transcription of genes typically required for differentiation. They are expressed in complex spatiotemporal patterns during embryonic development but their expression is commonly downregulated in mature tissues (reviewed in [5]).

Id1 is reported to be expressed in the luminal epithelium of the mammary gland during the early stages of mouse pregnancy [6] and to negatively regulate terminal differentiation of luminal epithelial cell lines *in vitro* [6], [7], [8]. However, there are no functional data addressing whether Id1 has a role in mammary development or differentiation *in vivo*. Furthermore, the validity of the antibody typically used in immunohistochemical studies of mammary Id1 expression (Santa Cruz SC-488) is disputed and some reports claim an absence of Id1 staining in the mammary gland [9], [10]. Id1 is also reportedly upregulated in breast cancer, with high expression correlating with poorer patient outcome [11]. Overexpression of Id1 promotes invasion, proliferation and migration *in vitro* [12], [13], [14] and high Id1 expression is associated with the metastatic phenotype of breast cancer cell lines *in vivo* [15]. We have previously shown that Id1 cooperates with oncogenic Ras in mammary tumourigenesis and metastasis *in vivo* [16], but the role for Id1 overexpression alone in mammary development and neoplasia has not been investigated.

Using a recently-developed monoclonal antibody we surveyed the expression of Id1 in the developing mouse mammary gland. We show that Id1 is not detected in the luminal epithelium at any timepoint during mammary development. To address the physiological role of Id1 in mammary development and neoplasia, we generated a transgenic mouse overexpressing Id1 under the control of the tetracycline regulatory element (TRE-Id1 strain). By breeding with mice expressing the reverse tetracycline transactivator (MTB strain) we generated a mouse with conditional expression of Id1 in the mammary gland. Based on the reported role of Id1 in preventing luminal differentiation *in vitro*, we predicted that these mice would possess dramatic defects in terminal mammary differentiation and lactation. However, we show that Id1 is not sufficient to prevent terminal mammary differentiation *in vivo* and these mice can undergo normal pubertal and pregnancy-associated mammary development.

## Results

### Expression of Id1 in the mammary gland

**Figure 1:**
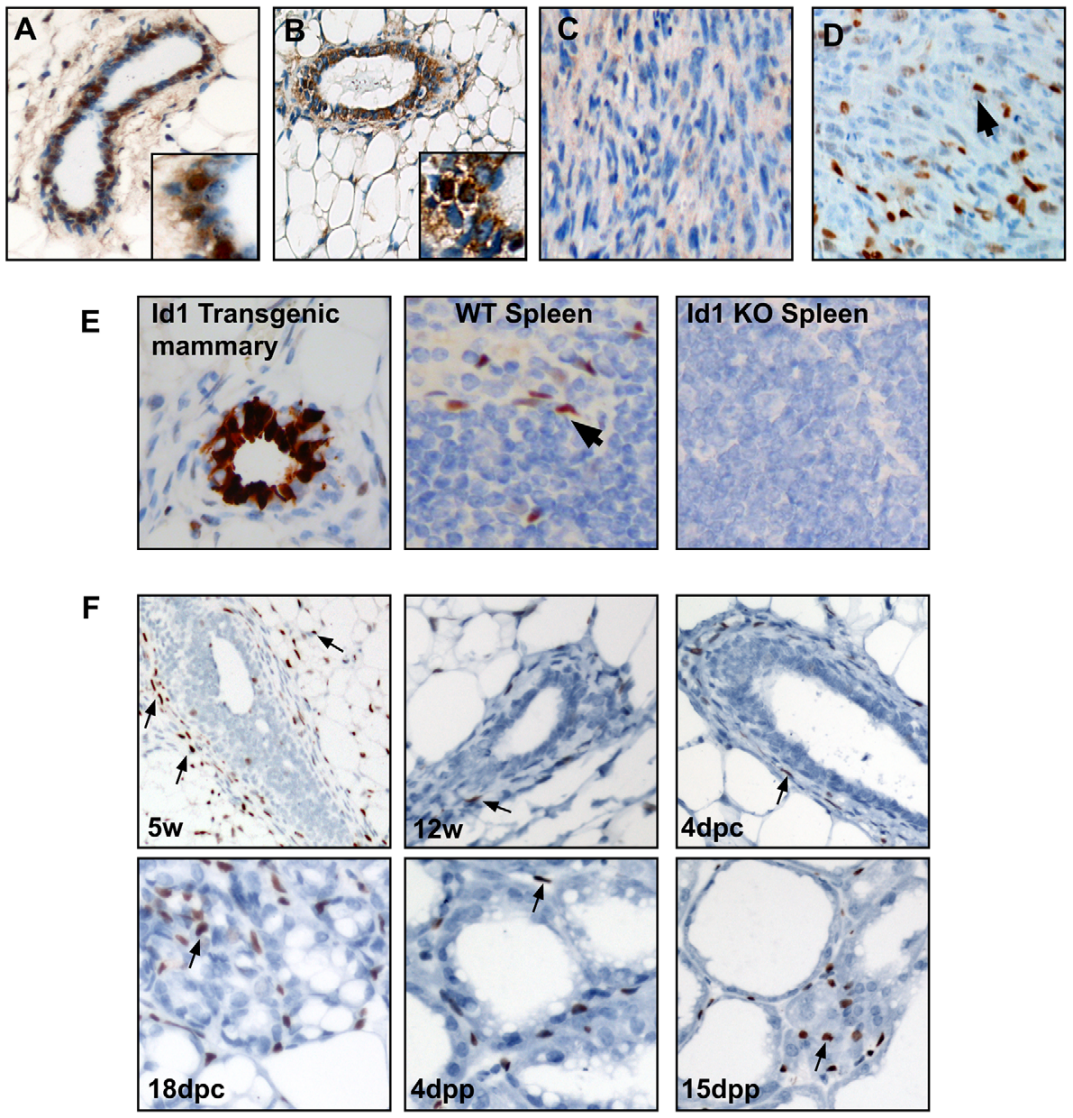
Id1 expression in the mouse mammary gland. Santa Cruz SC-488 polyclonal antibody does not specifically recognise Id1. Mammary glands from 10 week old virgin wildtype (A) and Id1-null (B) mice were immuno-stained for Id1 using SC-488. Note the non-specific positive staining of epithelial cells regardless of genotype (inset). A spontaneous p53-null mammary tumor was immunostained for Id1 using SC-488 (C) or BCH-1/#37-2 monoclonal antibody (D). (E) BCH-1/#37-2 monoclonal antibody was used to immunostain for Id1 in Id1-transgenic mammary glands, or spleen taken from a wildtype (WT) or Id1-null mouse. Id1-positive cells in the spleen are endothelium. (F) Id1 immunostaining (BCH-1/#37-2) was conducted on mouse mammary glands at various stages of mammary gland development: 5 weeks old, 12 week old virgin, 4 days post-coitus (dpc), 18 dpc, lactating 4 days postpartum (dpp) and lactating 15 dpp. Arrows indicate examples of Id1-positive cells.

To determine whether Id1 is normally expressed in the luminal epithelium during mammary development, as reported previously, we surveyed Id1 expression using a recently described monoclonal antibody to Id1 (Biocheck BCH-1/37-2; [9]) and compared it to the polyclonal antibody previously used to detect Id1 (SC-488; [6]). Staining with the polyclonal antibody was non-specific as positive nuclear and cytoplasmic staining was observed regardless of Id1 genotype (Figure 1A&B). The monoclonal antibody robustly detected Id1 in the mammary gland of bi-transgenic TRE-Id1+MTB animals as well as detecting endogenous Id1 expression in a proportion of cells in the mammary stroma and spleen of wildtype mice (Figure 1E). Staining of cells in the mammary stroma and spleen was absent in tissues from knockout hosts (Figure 1E). Staining with the monoclonal antibody BCH-1/37-2 did not readily detect Id1 expression in the mammary epithelium at any stage of mammary development, however nuclear Id1 expression was robustly detected in immune cells, endothelial cells and other stromal components (Figure 1F). Id1 was also not readily detected in the epithelium of normal human mammary gland derived from reduction mammoplasty (data not shown). We next used a spontaneous mouse model of basal-like breast cancer, derived from mammary transplants of p53 null epithelium, to test whether Id1 could be detected in mouse mammary tumours. Using the monoclonal antibody, Id1 positive cells were detected in tumours at a frequency ∼5–10% (Fig 1D). In comparison, the polyclonal antibody failed to detect Id1 positive cells (Figure 1C). These data demonstrate the high sensitivity and specificity of the monoclonal antibody compared to the low sensitivity and specificity of the polyclonal antibody.

### Generation of an Id1-transgenic mouse

**Figure 2:**
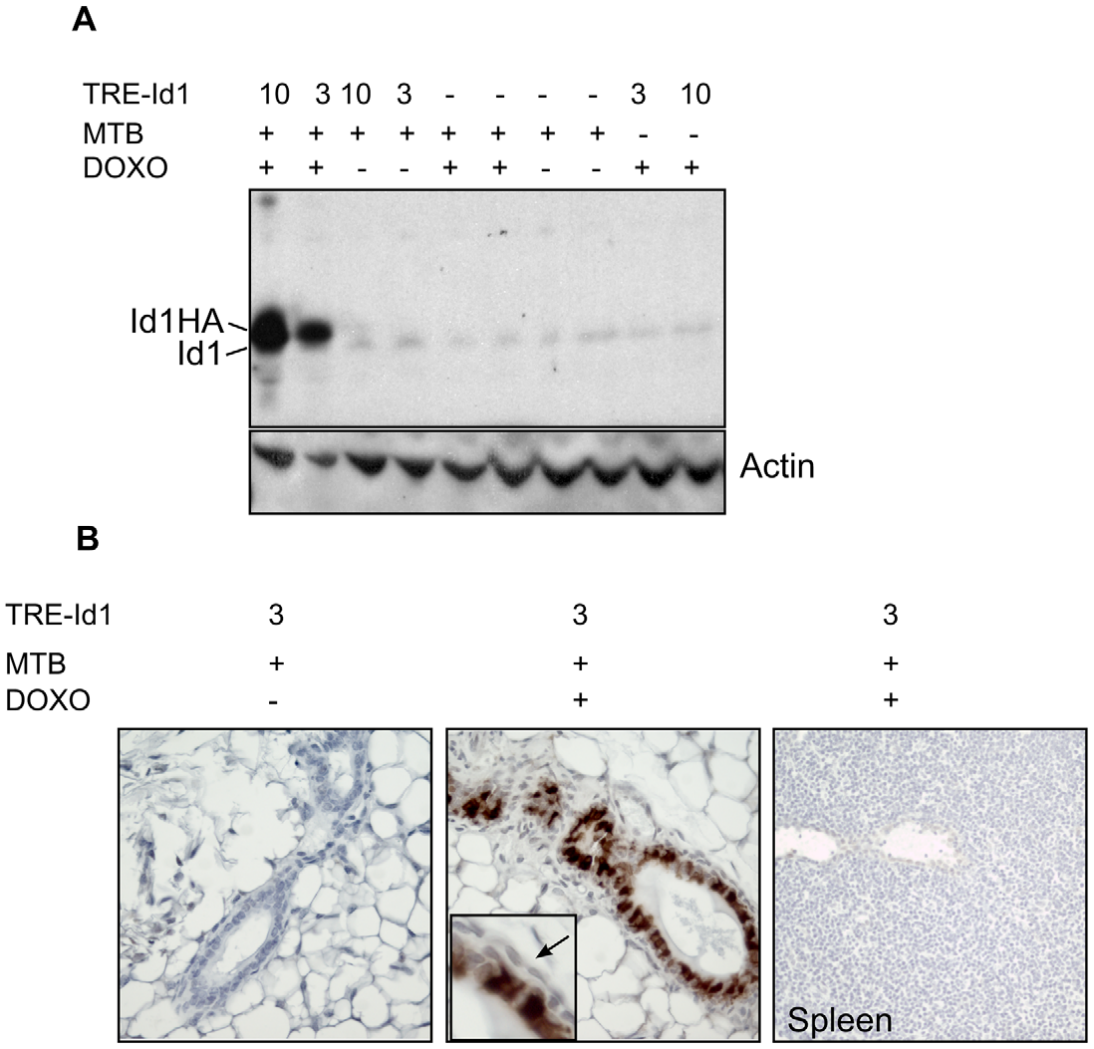
Generation of a mammary-specific conditional Id1-transgenic mouse. Mice from TRE-Id1 lines #3 or #10 were bred to MTB mice and administered dox chow for 5 days. (A) Mammary gland extracts were western blotted for Id1. The lower band is endogenous Id1 while the upper band corresponds to the Id1-HA transgene product. (B) Mammary glands or spleen were harvested and stained with anti-HA monoclonal antibody to demonstrate transgene expression in the luminal, but not in the myoepithelium (arrow) or non-epithelial organs such as spleen. TRE-Id1 line #10 gave identical results.

Extensive *in vitro* data (described earlier) suggests that Id1 controls luminal mammary epithelial cell fate and differentiation. Id1 was previously reported to be expressed in the mammary gland during the early stages of pregnancy, followed by a downregulation of Id1 concomitant with an upregulation of milk protein genes [6]. Id1 expression has also been shown to prevent terminal differentiation and production of milk proteins by immortalised mammary epithelial cells in culture [6], [7], [8], [17], [18]. While our earlier results suggested that Id1 is not expressed by luminal epithelia, it is possible that our histological analysis failed to identify a role for Id1 in luminal cell biology. Furthermore, since Id1 is expressed by breast cancers we wanted to test whether Id1 expression can initiate hyperplastic or neoplastic change in the mammary gland. To facilitate Id1 over-expression in the mammary gland, mice carrying a transgene encoding a hemaglutinin (HA) epitope-tagged Id1 cDNA downstream of the tetracycline response element promoter (TRE-Id1) were generated by pronuclear injection and crossed to mice carrying the MMTV-rtTA transgene (MTB; [19]). Two independent lines of TRE-Id1 mice were used for subsequent analysis. Id1 transgene expression was strongly induced in the mammary luminal epithelia of these mice by doxycycline addition *in vitro* and *in vivo* (Figure 2). In both transgenic lines, transgene expression was restricted to the luminal epithelium, as determined by immunohistochemical staining (Figure 2B, inset). There was no evidence of ‘leakiness’ in transgene expression in the absence of doxycycline, nor was the transgene expressed in unrelated tissues, such as the spleen, in the presence of doxycycline (Figure 2B). Representative data for both lines is shown in Figure 2B.

**Figure 3:**
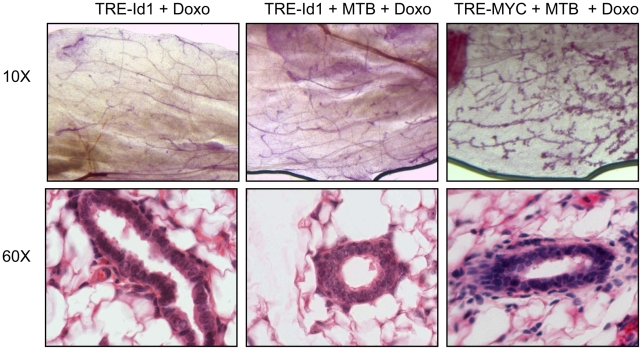
Ectopic Id1 expression is not sufficient to perturb virgin mammary gland development. Mice from TRE-Id1 lines #3 and #10 or TRE-Myc were bred to MTB mice and administered dox chow from 3 to 10 weeks of age to induce transgene expression. Mammary morphogenesis was analysed by carmine alum whole mounts (above) and by hematoxylin and eosin staining of sections from formalin-fixed, paraffin embedded glands (below). The histology of Id1 transgenic glands was unaffected by Id1 transgene expression during virgin mammary development, while control myc-transgenic glands display a hyperplastic phenotype. Images are representative of at least 5 mice per group, across two independent transgenic lines.

To examine the influence of Id1 expression during virgin mammary development, mice carrying the TRE-Id1 transgene alone or together with the MTB transgene were treated with doxycycline from weaning at 3 weeks of age to 9 weeks of age, so that Id1 was expressed throughout the period in which the mammary epithelium fills the fat pad and elaborates a mature ductal tree. Mice carrying the TRE-Myc and MTB transgenes were used as a positive control [20]. Using carmine-Alum staining of mammary gland whole mounts from these animals, there were no reproducible differences in ductal morphogenesis between TRE-Id1+MTB bi-transgenics and controls at this timepoint (Figure 3). Similarly, upon histological examination there was no reproducible effect on mammary epithelial morphology or stromal composition (Figure 3, Table S1). In comparison, overexpression of the c-Myc proto-oncogene caused an increase in ductal side-branching and hyperplastic morphology in the mammary gland (Figure 3).

**Figure 4:**
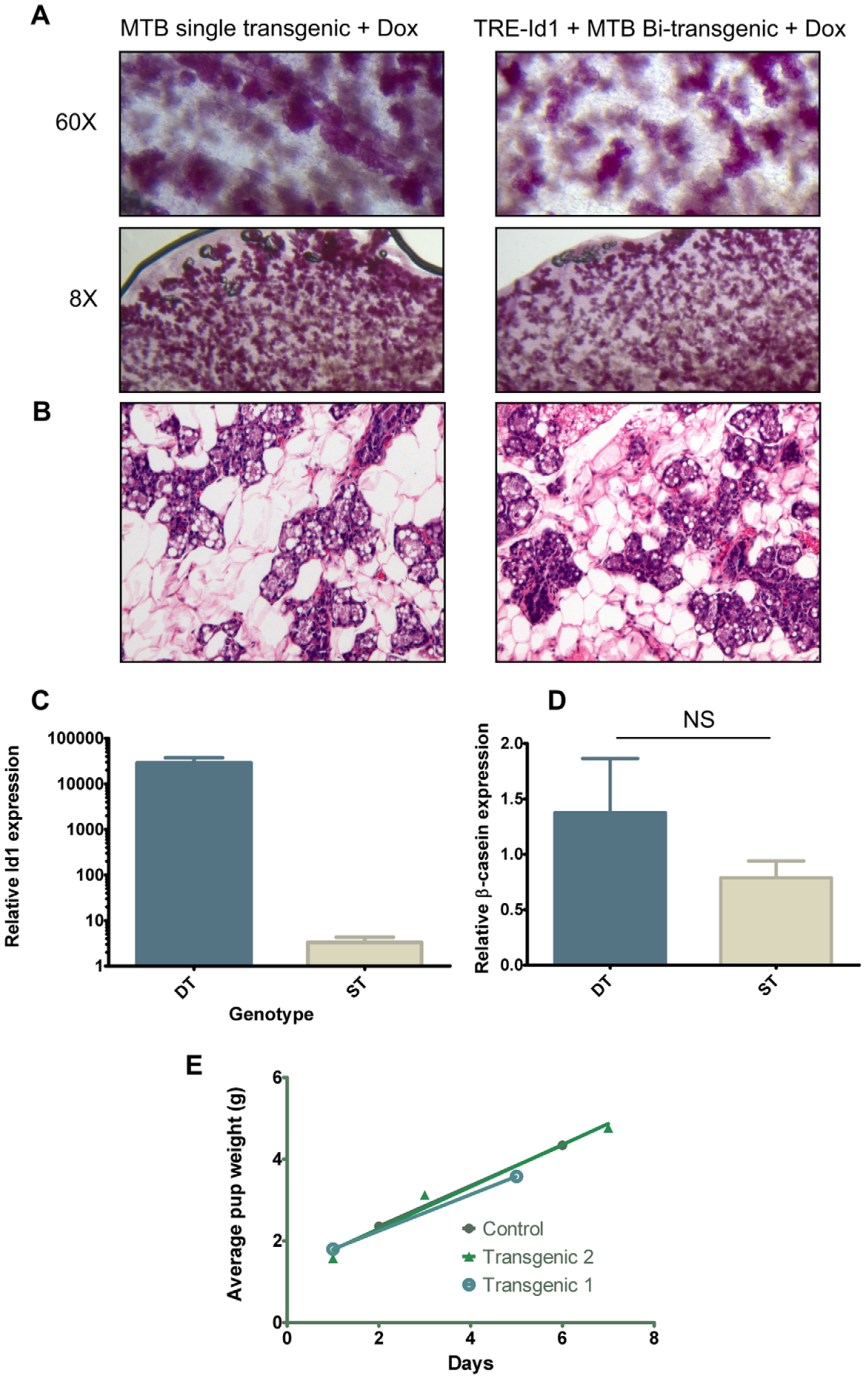
Id1 does not control terminal differentiation in the mammary gland. TRE-Id1/MTB double transgenic (DT) females or single transgenic (ST) controls were placed on dox chow from 6 weeks. (A–D) At ∼8–20 weeks, females were mated to wildtype males. At 16 days post coitus, mammary glands were harvested and analysed by carmine whole mount (A), sectioned for histology (B) or RNA extracted and analysed for Id1 (C) and β-casein gene expression (D). To determine whether TRE-Id1/MTB bitransgenic mice can lactate, pups were weighed after birth for up to 8 days and the average weight per litter compared to or single transgenic control mice (E).

During pregnancy, the mammary gland goes through rapid proliferation followed by entry into quiescence and terminal differentiation (reviewed in [21]). By day 9 of pregnancy, expression of milk proteins is induced and by day 16, WDNM1 and β-casein are widely expressed [22]. To determine whether expression of Id1 was incompatible with terminal mammary differentiation *in vivo*, Id1 expression was induced in bi-transgenic female mice and these mice were mated to FVB/N males. At 16 days of pregnancy, mammary glands were analysed for histology and gene expression. By whole mount (Figure 4A) and histology (Figure 4B) TRE-Id1+MTB bi-transgenic mammary glands were indistinguishable from those taken from similarly treated single transgenic control mice. Activation of milk protein expression was also unaffected, as β-casein expression was not significantly altered between Id1 overexpressing (Double transgenic; DT) and control glands (Single transgenic; ST; Fig 4D). To determine whether transgenic mice overexpressing Id1 were able to produce milk and feed pups, female bi-transgenic mice and single-transgenic controls were given doxycycline chow at the time of mating and pups observed. Milk was always observed in the stomach of pups from both experimental groups, and pups derived from mothers overexpressing Id1 grew at equivalent rates to those derived from control mothers (Fig 4E). Together, these data demonstrate that luminal Id1 expression does not control pubertal and pregnancy-associated mammary development nor prevent terminal differentiation of mammary epithelia.

## Discussion

Based on correlative analysis of Id1 expression during mammary development and experimentation with cell lines [6], [7], [8], Id1 has been proposed to regulate mammary differentiation and cell fate decisions. Using a highly sensitive and specific antibody we now provide definitive evidence that Id1 is not abundantly expressed in the mammary epithelium. Previous studies have reported Id1 expression in the mammary gland based on immuno-staining with a polyclonal antibody or by northern blotting of whole mammary extracts. We show that the polyclonal antibody has poor sensitivity and low specificity, and returns strongly positive immunostaining in both wildtype and Id1-null mammary glands. We detect Id1 expression in a number of stromal cell types, thus northern blotting of mammary extracts most likely detects Id1 expression in stromal cells rather than in the epithelium. However, Id1 may be expressed in rare epithelial cells within the mammary gland and we are currently investigating this possibility.

Id1 expression has previously been reported to correlate with poor prognosis in breast cancer [11], however that study used the polyclonal antibody that we report here to be non-specific and insensitive in mouse tissues. While we did not readily detect Id1 in the normal mammary epithelium, we did detect Id1 expression in a mouse mammary cancer model, and have similarly detected Id1 in human breast cancer cell lines and clinical cases (AS, unpublished data). This suggests that Id1 expression is activated during mammary neoplasia and that the prognostic significance of Id1 expression in breast cancer cohorts should be re-evaluated using this new monoclonal antibody, which we are currently pursuing.

Based on previous reports, we predicted that overexpression of Id1 in the luminal epithelial cells of the mammary gland would dramatically alter mammary development and pregnancy-related maturation. However, we demonstrate that Id1 expression alone is not sufficient to alter luminal epithelial cell fate nor to prevent terminal differentiation. Id1 transgenic mice underwent normal pubertal and pregnant mammary gland development, and were able to lactate and feed pups as normal. These data raise the question of why Id1 failed to regulate differentiation or mammary development. Unlike cells from control mice, cells taken from TRE-Id1 + MTB bi-transgenic mice were fully transformed by transduction with oncogenic h-Ras^V12^ expression (Figure S1) as previously reported [16], demonstrating that the Id1 transgene is active in these cells. The failure to regulate mammary development may therefore be a result of expression of the transgene in a non-physiologically relevant cell type, as we do not currently know whether the MMTV promoter directs transgene expression in the appropriate cell type in which Id1 is physiologically expressed. These results are consistent with a recent report that failed to detect a histological phenotype following Id1 transgene overexpression in the prostatic epithelium [23].

## Materials and Methods

### Generation and handling of mice

To determine the role for Id1 in mammary development and neoplasia *in vivo*, we generated a mouse carrying a transgene encoding murine Id1 cDNA under the control of the modified tetracycline response element, TREtight (Clontech). Linearised DNA encoding the transgene was injected into the pronuclei of FVB/N fertilized mouse oocytes by the UCSF transgenic core facility. Transgenic offspring were bred to FVB/N to establish two independent founder lines, named Id1#3 and Id1#10. Integration of the transgene was validated by southern blotting (data not shown) and expression was validated by harvesting tail fibroblasts, infecting with a retroviral construct encoding the tetracycline transactivator (tTA) and western blotting for Id1 (data not shown). MMTV-rtTA ([19];MTB) and TRE-Myc [24] mice were kindly provided by Dr Lou Chodosh (University of Pennsylvania). Mice were administered doxycycline by chow ad libitum (Bioserv Inc ;200 mg/kg). Experimental mice were treated according to protocol # 07/41 approved by the Institutional Animal Ethics Committee of the St Vincent's Hospital (Sydney) campus.

### Analysis of mammary gland and tumour specimens

For analysis of the effects of Id1 on pregnancy-induced mammary development, Id1 expression was induced in female nulliparous mice from ∼8 weeks of age, then at 10–12 weeks of age these mice were mated to FVB/N males and checked for plugs daily. After 16 days of pregnancy, mammary glands were collected and RNA was collected or glands were fixed and embedded into paraffin blocks.

RNA was prepared by Trizol (Invitrogen) extraction, followed by RT-PCR analysis for Id1, keratin 8 and β-casein using Taqman pre-designed gene expression assays (Applied Biosystems).

Mammary glands and mouse mammary tumours were fixed in 4% paraformaldehyde for 4–24 hours then transferred to 70% ethanol prior to processing and embedding in paraffin blocks. 4 µM sections were cut and stained either with hemotoxylin and eosin (H&E) or with antibodies to Id1 (Santa Cruz SC-488 or Biocheck clone BCH-1/37-2), HA (Santa Cruz SC-488), Cytokeratin 14 (Covance) using standard procedures. Whole mounts of transgenic mammary glands were also stained with Carmine Alum using a standard protocol. Histological analysis of mouse mammary glands was performed by a pathologists specialising in comparative pathology (ADB) blinded to the identity of each sample.

## Supporting Information

Figure S1 Mammary epithelial cells from TRE-Id1 X MTB transgenic mice are fully transformed by Ras activation. Epithelial cells from TRE-Id1 (line #3) X MMTV-rtTA mice were infected with a retrovirus encoding activated Ras, transplanted to the fat pad of four recipient mice and observed for 50 days. Aggressive tumours formed in all mice receiving these cells, and tumour diameters are shown. Wild-type epithelial cells transduced with Ras failed to form tumours. Preparation, infection and transplantation of cells was performed as previously described [16].
(5.00 MB TIF)

Table S1 Tabulation of histological analysis. Quantitation of histological analysis of 16 control (TRE-Id1 single transgenic + Dox) or 12 Id1-overexpressing mammary glands (TRE-Id1 X MMTV-rtTA + Dox) as described for Figure 3. A specialist veterinary pathologist (ADB) scored these as either (i) ‘normal-like’ or showing evidence of increase (ii) lobulogenesis or proliferation, (iii) ductal branching or (iv) a stromal reaction. The distribution of these phenotypes is similar between control and Id1 transgenic mice. Data from TRE-Id1 lines #3 and #10 have been combined.
(0.06 MB XLS)
