# Supplementary figures and images for: Redefining the Expression and Function of the Inhibitor of Differentiation 1 in Mammary Gland Development
Source: PLoS One. 2010 Aug 3;5(8):e11947. doi: 10.1371/journal.pone.0011947 (PMC2914758; doi:10.1371/journal.pone.0011947)

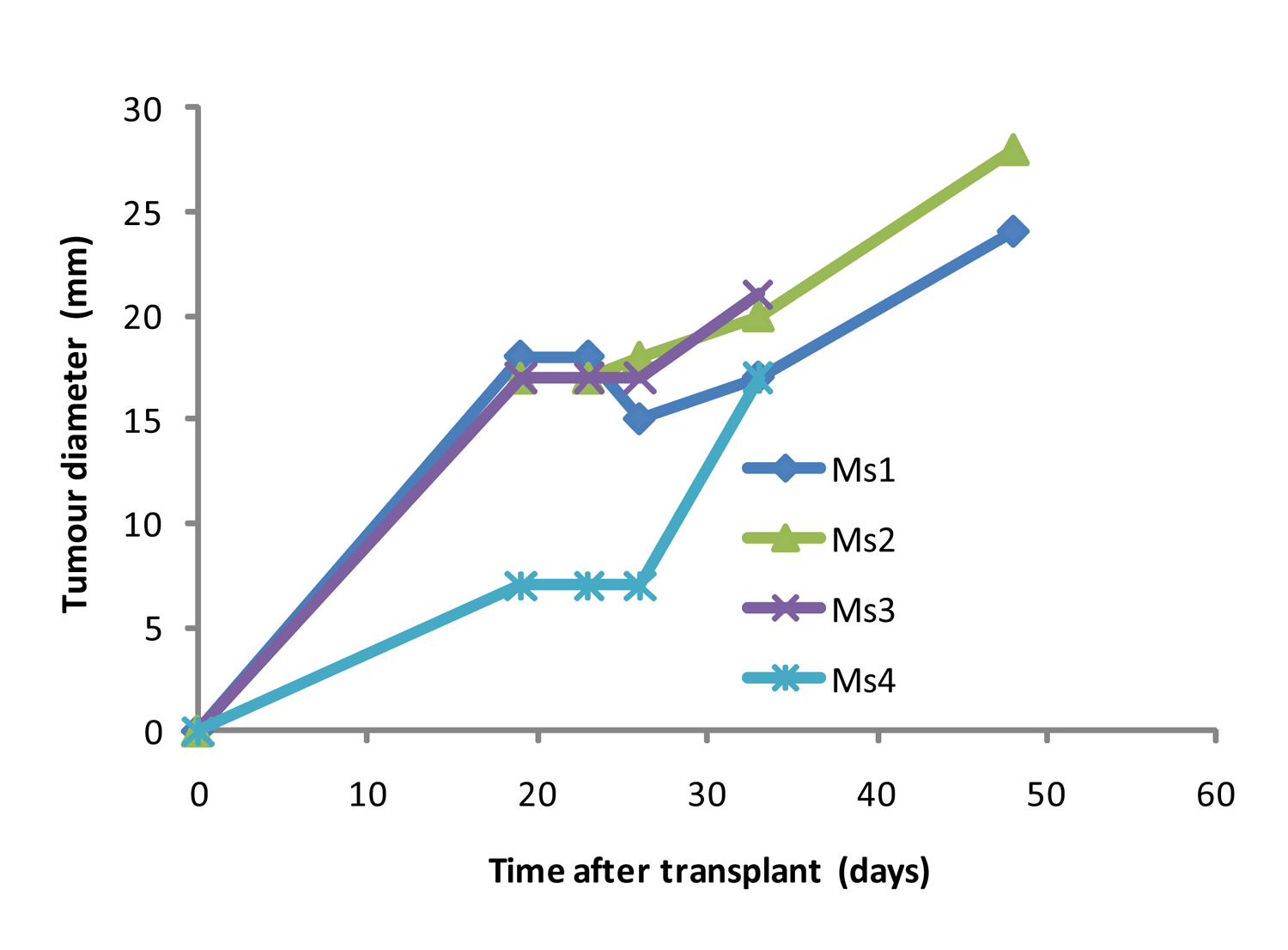

Supplement: Figure S1 — Mammary epithelial cells from TRE-Id1 X MTB transgenic mice are fully transformed by Ras activation. Epithelial cells from TRE-Id1 (line #3) X MMTV-rtTA mice were infected with a retrovirus encoding activated Ras, transplanted to the fat pad of four recipient mice and observed for 50 days. Aggressive tumours formed in all mice receiving these cells, and tumour diameters are shown. Wild-type epithelial cells transduced with Ras failed to form tumours. Preparation, infection and transplantation of cells was performed as previously described [16]. (5.00 MB TIF) [file pone.0011947.s001.tif]
